# Measurement of Nanomolar Dissociation Constants by Titration Calorimetry and Thermal Shift Assay – Radicicol Binding to Hsp90 and Ethoxzolamide Binding to CAII

**DOI:** 10.3390/ijms10062662

**Published:** 2009-06-10

**Authors:** Asta Zubrienė, Jurgita Matulienė, Lina Baranauskienė, Jelena Jachno, Jolanta Torresan, Vilma Michailovienė, Piotras Cimmperman, Daumantas Matulis

**Affiliations:** Laboratory of Biothermodynamics and Drug Design / Institute of Biotechnology, Graičiūno 8, Vilnius, LT-02241, Lithuania; E-Mails: astzu@ibt.lt (A.Z.); matuliene@ibt.lt (J.M.); linami@ibt.lt (L.B.); elper@ibt.lt (J.J.); jvanagel@ibt.lt (J.T.); roze@ibt.lt (V.M.); piotras@ibt.lt (P.C.)

**Keywords:** Hsp90, radicicol, thermal shift assay, isothermal titration calorimetry, protein-ligand binding, carbonic anhydrase, ethoxzolamide

## Abstract

The analysis of tight protein-ligand binding reactions by isothermal titration calorimetry (ITC) and thermal shift assay (TSA) is presented. The binding of radicicol to the *N*-terminal domain of human heat shock protein 90 (Hsp90αN) and the binding of ethoxzolamide to human carbonic anhydrase (hCAII) were too strong to be measured accurately by direct ITC titration and therefore were measured by displacement ITC and by observing the temperature-denaturation transitions of ligand-free and ligand-bound protein. Stabilization of both proteins by their ligands was profound, increasing the melting temperature by more than 10 ºC, depending on ligand concentration. Analysis of the melting temperature dependence on the protein and ligand concentrations yielded dissociation constants equal to 1 nM and 2 nM for Hsp90αN-radicicol and hCAII-ethoxzolamide, respectively. The ligand-free and ligand-bound protein fractions melt separately, and two melting transitions are observed. This phenomenon is especially pronounced when the ligand concentration is equal to about half the protein concentration. The analysis compares ITC and TSA data, accounts for two transitions and yields the ligand binding constant and the parameters of protein stability, including the Gibbs free energy and the enthalpy of unfolding.

## 1. Introduction

When a ligand binds to a protein with a single-digit nanomolar or tighter dissociation constant, it is difficult to measure the affinity accurately using isothermal titration calorimetry (ITC) because the titration curve becomes too steep to fit accurately. In such cases, one can measure the displacement of a weakly binding ligand during titration with a strongly binding ligand of interest [1,2]. However, this method requires weakly binding ligands with accurately determined binding constants. There are a number of factors increasing the error of measurements in the displacement assay.

Here, we discuss an alternative method to determine the binding constants of strongly binding ligands – the thermal shift assay – that could be used in addition to the displacement assay. The thermal shift assay (TSA) [3], also known as differential scanning fluorimetry [4] and ThermoFluor® [5], is a high-throughput screening method for hit selection and determination of protein-ligand binding constants used in the pharmaceutical industry [6]. This biophysical technique can be applied to any protein-ligand noncovalent binding reaction, and is independent of whether the ligand stabilizes or destabilizes the protein upon binding [7]. In addition, the method is useful for the characterization of protein stability in the presence of various excipients [8] and the optimization of conditions for protein crystallization [9].

The phenomenon of protein stabilization against thermal denaturation by ligands was shown to be a useful method for determining ultratight binding constants [10]. It was originally carried out by differential scanning calorimetry (DSC) [11] and later by measuring the increase in fluorescence of a hydrophobic probe, 1,8-anilinonaphthalene sulfonate (ANS). ANS fluorescence in an aqueous solution is quenched by water and increases upon protein unfolding due to thermal denaturation and the resulting opening of hydrophobic pockets that prevent water quenching. The phenomenon was first discovered by [12,13] and reviewed by [14]. The mechanism of ANS binding to proteins involves ion pair formation between ANS sulfonate groups and positively charged amino acid residues [15,16]. However, the ion pair formation is not visible by fluorescence and, therefore, does not affect the observed denaturation profile.

An investigation using a combination of DSC, ITC, and TSA of aromatic sulfonamide inhibitor binding to carbonic anhydrase yielded a full thermodynamic picture of the coupled denaturation and binding reactions [17]. Consistency of the thermodynamic parameters obtained using all three methods is important to obtain a full thermodynamic description of the protein-ligand interaction. However, the application of TSA to the measurement of radicicol binding to heat shock protein 90 (Hsp90) yielded atypical dose-response curves [18]. Similar curves were observed when measuring ethoxzolamide binding to carbonic anhydrase. When the concentration of ligand was lower than that of protein, two unfolding transitions were observed – the first due to unfolding of the unbound protein and the second due that of the protein-ligand complex. Similar biphasic denaturation behavior has been previously observed using DSC with human serum albumin at subsaturating concentrations of a tightly binding ligand [11,19,20]. To account for the two transitions, a formula that allows the determination of the ligand-protein binding constant is described in this work.

Hsp90 is a molecular chaperone responsible for the correct folding of a large number of proteins. It is one of the most abundant proteins in eukaryotic cells, comprising 1–2% of the cellular protein content under non-stress conditions. Hsp90 is highly conserved from bacteria to humans. In cancerous cells, Hsp90 is essential for tumor progression because the Hsp90 machinery helps to maintain numerous altered or overexpressed client proteins in their active forms. Since multiple oncogenic proteins can be simultaneously degraded upon inhibition of Hsp90 by small molecule inhibitors, Hsp90 has evolved into a promising anticancer target. Various aspects of Hsp90 have been reviewed [21], including its biology [22,23], inhibitors [24], and potential as an anticancer treatment target [25–27]. The structure of the entire yeast Hsp90 dimeric complex with p23 was determined by X-ray crystallography, providing further insight into the mechanism of Hsp90 [28].

Radicicol, also known as monorden, was originally discovered as an antifungal substance of fungal origin in 1953 [29] and is a specific Hsp90 inhibitor [30]. The co-crystal structure of radicicol bound to yeast Hsp90N was determined, and showed numerous hydrogen bonding interactions between radicicol and the ATP-binding pocket of the N-terminal domain [31]. Radicicol, as determined by isothermal titration calorimetry (ITC), is a tight binder with a dissociation constant (*K_d_*) of the order of 1 nanomolar [31]. Full-length yeast Hsp90 bound to radicicol with a *K_d_* of 19 nM, and the N-terminal domain (1–220 a.a.) of yeast Hsp90 bound to radicicol with a *K_d_* of 2.7 nM. As pointed out by the authors, the binding was too tight to be determined directly by ITC. Here, we determined the binding constant of radicicol to the N-terminal domain of human Hsp90 using the thermal shift assay. It is important to determine the thermodynamics of radicicol binding to human Hsp90 since this natural compound is widely used as a model of inhibitors with anticancer properties [32].

Carbonic anhydrases (CAs; EC 4.2.1.1) are ubiquitous zinc-metalloenzymes that catalyze the conversion of CO_2_ to bicarbonate. In humans, 15 different α-CA isozymes have been described, 12 of which have catalytic activity [33]. Many of the CA isozymes are important therapeutic targets – their inhibition is used for treatment of diseases such as glaucoma, edema, epilepsy, osteoporosis, and others [34]. Human CAII is a cytoplasmic isozyme detected in almost all tissues and organs, and it is one of the fastest enzymes known with a CO_2_ hydration turnover number *k_cat_*=10^6^ s^−1^ [35].

Ethoxzolamide is a drug previously used as an antiglaucoma and antibacterial agent and for the treatment of edema. It is one of the strongest known inhibitors of carbonic anhydrase (hCAII *K_i_*=8 nM). Recently, the structure of the hCAII-ethoxzolamide complex was solved to 1.80 Å, and the structural details of the extremely tightly bound complex were revealed [36]. Here, we determine the binding constant of ethoxzolamide to hCAII using the thermal shift assay.

The thermal shift assay method can be applied to atypical dose curves of extremely tight binding reactions when the use of ITC requires displacement titration. The TSA method is limited only by the boiling temperature of water. The method can be used in miniaturized and high-throughput plate formats for almost any protein-ligand binding reaction. Therefore, it could serve as a primary option for the determination of protein-ligand binding constants.

## 2. Results and Discussion

### 2.1. Derivation of the 2-stage thermal shift assay binding model

The fluorescence intensity of the probe ANS is dependent on the molecular environment of the anilinonaphthalene group. Upon protein unfolding, a few ANS molecules bind through a combination of hydrophobic and ionic [16] forces to the protein interior, preventing quenching and thus increasing fluorescence. Remaining ANS anions are free in the aqueous solution or are bound to the surface of the protein, and thus are exposed to and quenched by water. Therefore, they are invisible by fluorescence.

The probability of the protein unfolding can be described by the equation:

(1) $$P_{U}=\frac{1}{1+e^{\Delta_{U}G/RT}}$$

where Δ*_U_G* is the Gibbs free energy of unfolding, *R* the universal gas constant, and *T* the absolute temperature. The Gibbs free energy of unfolding can be expressed in terms of the enthalpy (Δ *_U_H*), entropy (Δ*_U_S*), and heat capacity (Δ*_U_C_p_*) change of protein unfolding:

(2) $$\Delta_{U}G=\Delta_{U}H_{T_{m}}+\Delta_{U}C_{p}(T-T_{m})-T(\Delta_{U}S_{T_{m}}+\Delta_{U}C_{p}\;\text{ln}(T/T_{m}))$$

where the enthalpy and entropy are at *T_m_* while the heat capacity is assumed to be temperature-independent in the studied temperature range.

The ANS fluorescence intensity in the protein denaturation curve can be described by the equation:

(3) $$y(T)=y_{U}P_{U}+y_{F}\;(1-P_{U})$$

where *y_F_* and *y_U_* are the temperature dependent fluorescence intensities in the presence of native (folded) and unfolded protein, respectively. 1–*P_U_* is the probability of the protein to be in the folded state. When the ligand-bound and ligand-free protein fractions denature independently, the fluorescence intensity in the protein denaturation curve has the following form:

(4) $$y(T)=n(y_{U\_bound}P_{U\_bound}+y_{F\_bound}(1-P_{U\_bound}))+(1-n)(y_{U\_free}P_{U\_free}+y_{F\_free}(1-p_{U\_free}))$$

where the indices “bound” and “free” are used to identify the parameters of ligand-bound and ligand-free protein, respectively. The parameter *n* denotes the fraction of ligand-bound protein and varies from 0 to 1.

Since the fluorescence intensities of proteins with and without bound ligand are equal for both the folded and unfolded protein forms, the following simplification is made:

(5) $$\begin{array}{c} y_{F\_free}=y_{F\_bound}=y_{F};\\ y_{U\_free}=y_{U\_bound}=y_{U}; \end{array}$$

After applying Equation (5), Equation (4) is rearranged to:

(6) $$y(T)=y_{F}+(y_{U}-y_{F})((1-n)P_{U\_free}+nP_{U\_bound})$$

Combining Eqs. (1), (2), and (6), the temperature dependence of the fluorescence for two transitions (ligand-free and ligand-bound protein whose relative fractions depend on the ligand concentration) is obtained:

(7) $$\begin{array}{c} y(T)=y_{F}+(y_{U}-y_{F})(\frac{1-n}{1+e^{(\Delta_{U}H_{free}+\Delta_{U}C_{p}(T-T_{free})-T(\Delta_{U}S_{free}+\Delta_{U}C_{p}\;\text{ln}\;(T/T_{free})))/RT}}+\\ \;\;\;\;\;\frac{n}{1+e^{(\Delta_{U}H_{bound}+\Delta_{U}C_{p}(T-T_{bound})-T(\Delta_{U}S_{bound}+\Delta_{U}C_{p}\;\text{ln}\;(T/T_{bound})))/RT}}) \end{array}$$

where *T_free_* is the protein melting temperature in the absence of ligand and *T_bound_* is the protein melting temperature in the presence of ligand. The thermodynamic parameters of unfolding, the enthalpy and entropy, were designated with the subscripts ‘free’ or ‘bound’ to represent the two protein forms.

The fluorescence of ANS before and after the transition depends on the temperature. Since the ANS fluorescence has curvature as a function of temperature, the fluorescence dependence on temperature (in the absence of the protein unfolding transition) is accurately represented by the quadratic polynomial:

(8) $$y_{F}=a_{F}+b_{F}(T-T_{ref})+c_{F}{\left(T-T_{ref}\right)}^{2}$$

(9) $$y_{U}=a_{U}+b_{U}(T-T_{ref})+c_{U}{\left(T-T_{ref}\right)}^{2}$$

where *a_F_* and *a_U_* are fluorescence intensities of ANS in the presence of native and unfolded protein, respectively, at the temperature *T_ref_*, while *b* and *c* are empirically determined coefficients for folded and unfolded protein forms. The coefficients *b* and *c* were determined by the global fit of the experimental data and kept constant among different melting curves. *T_ref_* is a freely chosen temperature (in this case 0 ºC).

The final formula describing the temperature dependence of fluorescence is obtained by substituting Equation (8) and Equation (9) into Equation (7):

(10) $$\begin{array}{l} y(T)=a_{F}+b_{F}\;(T-T_{ref})+c_{F}{\left(T-T_{ref}\right)}^{2}+(a_{U}-a_{F}+(b_{U}-b_{F})(T-T_{ref})+(c_{U}-c_{F}){\left(T-T_{ref}\right)}^{2})\times \\ \;\;\;\;\;\;\;\;\;\left(\frac{1-n}{1+e^{(\Delta_{U}H_{free}+\Delta_{U}C_{p}(T-T_{free})-T(\Delta_{U}S_{free}+\Delta_{U}C_{p}\;\text{ln}\;(T/T_{free})))/RT}}+\frac{n}{1+e^{(\Delta_{U}H_{bound}+\Delta_{U}C_{p}(T-T_{bound})-T(\Delta_{U}S_{bound}+\Delta_{U}C_{p}\;\text{ln}\;(T/T_{bound})))/RT}}\right) \end{array}$$

Equation (10) was fit to a family of experimental curves obtained at various protein and ligand concentrations, yielding the melting temperatures of both the free and bound protein forms. The standard deviation of the melting temperature was approximately 0.3 ºC.

In previous studies [7,17], we described the derivation of an equation relating the total ligand concentration (*L_t_*) needed to raise the protein melting temperature to a certain value depending on the thermodynamics of protein unfolding and ligand binding:

(11) $$L_{t}=(K_{U\_T_{m}}-1)\left(\frac{P_{t}}{2K_{U\_T_{m}}}+\frac{1}{K_{b\_T_{m}}}\right)$$

where *P_t_* is the total added protein concentration and *K_U_T_m__* and *K_b_T_m__* are the protein unfolding and ligand binding equilibrium constants at the temperature *T_m_*, respectively. Equation (11) can be expressed in terms of the enthalpy, entropy, and heat capacity of unfolding and binding:

(12) $$\begin{array}{l} L_{t}=\left(e^{-(\Delta_{U}H_{T_{r}}+\Delta_{U}C_{p}(T_{m}-T_{r})-T_{m}(\Delta_{U}S_{T_{r}}+\Delta_{U}C_{p}\text{ln}(T_{m}/T_{r})))/RT_{m}}-1\right)\\ \;\times \left[\frac{P_{t}}{2}\frac{1}{e^{-(\Delta_{U}H_{T_{r}}+\Delta_{U}C_{p}(T_{m}-T_{r})-T_{m}(\Delta_{U}S_{T_{r}}+\Delta_{U}C_{p}\;\text{ln}(T_{m}/T_{r})))/RT_{m}}}+\frac{1}{e^{-(\Delta_{b}H_{T_{0}}+\Delta_{b}C_{p}(T_{m}-T_{0})-T_{m}(\Delta_{b}S_{T_{0}}+\Delta_{b}C_{p}\text{ln}(T_{m}/T_{0})))/RT_{m}}}\right] \end{array}$$

where *U* and *b* represent the unfolding and binding parameters, respectively. *T*_0_ is the reference temperature of the binding reaction, usually 37 ºC. The temperature *T_r_* is the reference temperature of protein melting without added ligand.

### 2.2. Radicicol binding to Hsp90 and ethoxzolamide binding to hCAII by titration calorimetry

**Figure 1.**
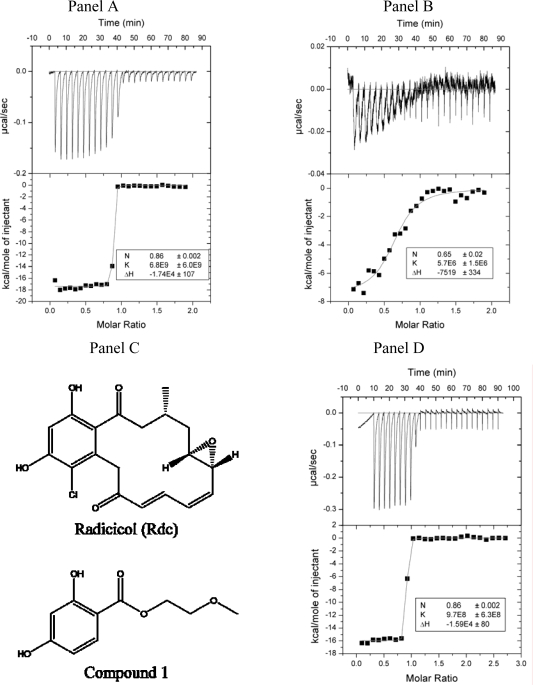
Panel A. Isothermal titration calorimetry data from radicicol binding to Hsp90αN. Upper graph – raw ITC data, lower graph – integrated ITC data with the curve fit to the standard single binding site model. The cell contained 4 μM protein, while the syringe contained 40 μM radicicol in the same buffer - 50 mM sodium phosphate, pH 7.5, 0.5% DMSO, 100 mM NaCl, at 25 °C. Panel B. Isothermal titration calorimetry displacement assay. All conditions are the same as in Panel A, except there was 1 mM compound 1 added to the calorimeter cell. Panel C. Chemical structures of radicicol and compound 1. Panel D. Isothermal titration calorimetry data from ethoxzolamide binding to hCAII. The cell contained 7 μM protein, while the syringe contained 100 μM ethoxzolamide in the same buffer - 50 mM sodium phosphate, pH 7.0, 0.5% DMSO, 50 mM NaCl, at 37 °C.

Isothermal titration calorimetry measurements of radicicol binding to Hsp90 constructs and ethoxzolamide binding to hCAII yielded very steep binding curves (Figure 1A, 1D). Such curves are too steep to fit binding constants accurately. To obtain valid binding constants, the *c* factor must be between 5 and 500: *c* =*n*[*M*]*K_b_*, where *n* is the binding stoichiometry, [M] the macromolecule concentration, and *K_b_* the binding constant). Fitting the data in Figure 1A yields the *K_b_*=7 × 10^9^ M^−1^. Numerous repeats of this experiment yielded a binding constant in the range of 10^8^ to 10^11^ M^−1^. Since the binding stoichiometry is 1 and the protein concentration is 4 μM, the c factor in Figure 1A is 28,000. Therefore, the binding constant is significantly beyond the upper limit for accurate determination by ITC. Reduction of protein and ligand concentrations is not feasible since the data to noise ratio becomes too low.

Tight ligand binding constants can be determined by carrying out the displacement of a weakly binding ligand [2]. Radicicol binding experiments were carried out in the same conditions as in Figure 1A except for the addition of 1 mM compound 1 to the calorimeter cell (Figure 1B, chemical structure in Figure 1C). The apparent binding constant was 6 (±2) × 10^6^ M^−1^. The binding constant of compound 1 was determined to be 2 (±1) × 10^4^ M^−1^. The true binding constant can be determined by:

$$K_{b}=K_{b-\text{apparent}\;}(1+K_{b-\text{compound}\;1}[\text{compound}\;1])=6\times 10^{6}\times (1+2\times 10^{4}\times 10^{-3})=1\times 10^{8}{\text{M}}^{-1}$$

This value is approximate and significantly lower than the value obtained by the direct ITC experiment. Additional methods were needed in order to determine the compound binding constants with greater accuracy.

### 2.3. Radicicol binding to Hsp90 and ethoxzolamide binding to hCAII by TSA and DSC

The *N*-terminal domain of human Hsp90 (Hsp90αN) denatures upon heating, with the midpoint of its unfolding transition occurring at approximately 52–53 ºC. The exact melting temperature depends on the Hsp90αN concentration and is equal to 51.6 ºC at 14 μM (Table 1) and 52.7 ºC at 7.5 μM.

These results are similar to the melting of Hsp90 from other organisms. For example, A. Makarov and coworkers determined the transition midpoint of the *N*-terminal domain of Hsp90 from porcine brain to occur at 53.8 ºC by DSC and 53.3 ºC by circular dichroism [37].

**Figure 2.**
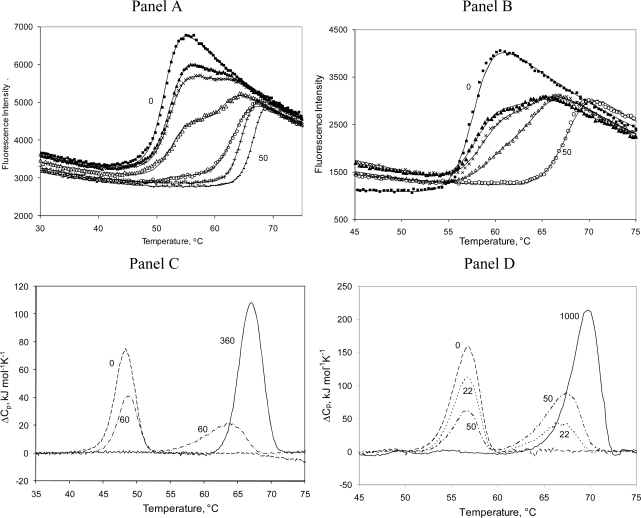
Denaturation profiles of Hsp90αN or hCAII in the presence of inhibitors. Panel A. Hsp90αN (14 μM) in 50 mM Tris buffer, pH 7.5, with added radicicol: ▪ - 0 μM, ▴ - 2 μM, × - 3 μM, Δ - 6 μM, ○ - 10 μM, + - 20 μM, and − - 50 μM. Panel B. hCAII (5 μM) in 50 mM phosphate buffer, pH 7.0, containing 50 mM NaCl, with added ethoxzolamide: ▪ - 0 μM, ▴ - 1.5 μM, × - 2 μM, Δ - 3 μM, and ○ - 50 μM. Increased inhibitor concentrations raise the melting temperature of both proteins by more than 10 ºC, depending on concentration. Two transitions are observed when both free and ligand-bound proteins are present. Heights of both transitions are additive and proportional to the fraction of saturation by the inhibitor. The data points represent experimental observations while the lines are fit to Equation (10). Parameters are listed in Table 1. Panel C. DSC profiles of Hsp90αN (120 μM) with radicicol: dashed line – 0 μM, dot-dashed line – 60 μM, and solid line – 360 μM. Panel D. DSC profiles of hCAII (100 μM) with ethoxzolamide: dashed line – 0 μM, dotted line – 22 μM, dot-dashed line – 50 μM, and solid line – 1 mM.

**Figure 3.**
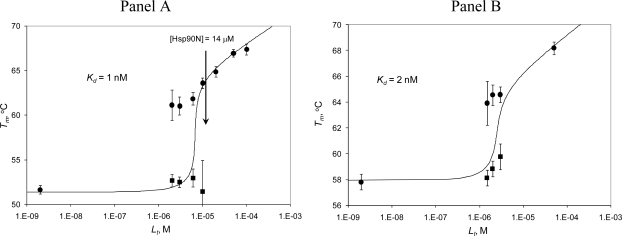
The protein melting temperature dependence on inhibitor concentration. Panel A. Hsp90αN with radicicol (from the melting curves in Figure 2A). Panel B. hCAII with ethoxzolamide (from the melting curves in Figure 2B). The data points show the melting temperatures of the ligand-bound (•) and ligand-free (▪) forms of the protein. The leftmost data point is the control where no inhibitor was added. Lines are the fit to the data according to Equation (12).

Radicicol specifically binds to the ATP site of the N-terminal domain of Hsp90 with a stoichiometry of one radicicol molecule bound to each Hsp90 monomer. The addition of radicicol dramatically increases the Hsp90αN melting temperature by 15 ºC or more, depending on the ligand concentration (Figures 2A and 3A). For example, the addition of 50 μM radicicol raised the melting temperature of Hsp90αN from 51.6 to 66.9 ºC at 14 μM protein.

The addition of radicicol concentrations greater than the protein concentration yielded a single melting transition. However, addition of concentrations of radicicol lower than the protein concentration yielded a profile that is split into two melting transitions. The first transition, the melting transition of free Hsp90αN, occurred at a lower temperature with the temperature midpoint being essentially independent of the ligand concentration (Table 1). The second transition was the melting transition of the Hsp90αN-radicicol complex. The temperature midpoint of the second transition was strongly dependent on radicicol concentration.

There is a possibility, raised in the literature, that radicicol binds covalently and irreversibly to the protein [18]. In the presence of a high concentration of reducing agents such as dithiothreitol (DTT), radicicol was shown to be unstable and was covalently modified [38]. To demonstrate that there is no covalent modification of Hsp90 by radicicol in our experimental conditions, protein solutions with and without added radicicol were analyzed by mass spectrometry. The molecular weight (MW) of the protein in the absence of radicicol was determined to be 31,270.6 Da. This is in good agreement with the theoretical MW calculated from the amino acid sequence, 31,270.80 Da. The MW of the protein incubated with radicicol at a 10:1 radicicol to protein molar ratio under the same conditions yielded a value between 31,270.5 and 31,270.8 Da. It was concluded that no covalent modification of Hsp90 occurs in the protein-radicicol solution.

Similar splits of ligand-bound and free denaturation transitions were observed with ethoxzolamide binding to recombinant human carbonic anhydrase II. Figure 2B shows the denaturation profiles from the thermal shift assay, and Figures 2C and 2D demonstrate the same split into two transitions observed by differential scanning calorimetry for both proteins. Increasing the ligand concentration diminishes the first peak and increases the second peak proportionally to the ligand-bound fraction of the protein. When the protein is saturated with the ligand, the first transition disappears.

Curves in Figures 2A and 2B were fit globally to the model of Equation (10). The fitting parameters are listed in Table 1, including the enthalpies of denaturation of the free and ligand-bound forms of both proteins. There was a large increase in the unfolding enthalpy due to the positive heat capacity of protein unfolding. For example, the enthalpy of Hsp90αN unfolding as determined by TSA was 670 kJ/mol at 51.6 ºC and 900 kJ/mol at 66.9 ºC, while the values determined using DSC were 290 kJ/mol at 48.2 ºC and 477 kJ/mol at 66.9 ºC. Similarly, the enthalpy of hCAII unfolding measured by TSA was 690 kJ/mol at 57.8 ºC and 780 kJ/mol at 68.2 ºC, while the enthalpy of hCAII unfolding determined by DSC was 570 kJ/mol at 56.5 ºC and 790 kJ/mol at 69.4 ºC in the presence of 1 mM ethoxzolamide. There is approximate match between the TSA and DSC enthalpies for hCAII, but the enthalpies of Hsp90αN are significantly smaller as measured by DSC compared to the values obtained using TSA. The DSC values are likely to be more accurate than the TSA values.One can estimate from the enthalpy of unfolding values determined above that the heat capacity of Hsp90αN unfolding is about 15 kJ×mol^−1^×K^−1^ by TSA and 10 kJ×mol^−1^×K^−1^ by DSC, while the heat capacity values for hCAII are 17 kJ×mol^−1^×K^−1^ and 9 kJ×mol^−1^×K^−1^ by DSC and TSA, respectively. These numbers are approximate but similar to the values of 13.8 kJ×mol^−1^×K^−1^ for Hsp90αN, which was calculated from a set of 49 protein Δ*_U_C_p_* values correlating with the number of residues in the protein (58 J×mol-residue^−1^×K^−1^) [39], and 15.9 kJ×mol^−1^×K^−1^ for bCAII based on the DSC data [17]. However, it should be emphasized that the heat capacity values are approximate and are not well determined by the thermal shift assay.

Figure 3 shows the split of the protein melting transition into two melting temperatures, plotted as a function of added ligand concentration. Similar plots were drawn by representing linkage in phase analysis [40]. Data points represent experimentally obtained data, while the lines are fits to the model described by Equation (12). The leftmost data point is obtained in the absence of radicicol (Figure 3A) or ethoxzolamide (Figure 3B). The model described in [17] averages the two transitions into a single transition at a temperature dependent on ligand concentration. Here we fit the two transitions at all ligand concentrations that are lower than that of the protein. The transition of free protein occurs at essentially the same temperature, independent of ligand concentration. When the ligand nearly saturates the protein, the error increases and the transition finally disappears. Similarly, the transition of bound protein is poorly visible at very low ligand concentrations, when the bound form of the protein is a minor component. When the ligand concentration is increased, the transition of the bound form becomes dominant and eventually masks the transition of free protein. The second ligand-bound transition depends on ligand concentration and follows the model of Equation (12) yielding the dissociation constants equal to *K_d_*=1 nM (Hsp90αN) and 2 nM (hCAII).

**Figure 4.**
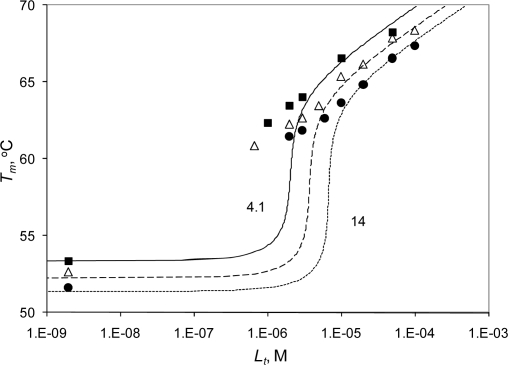
The dependence of the melting temperature of Hsp90αN on radicicol concentration. The data points show the melting temperatures of ligand-bound components at three protein concentrations: ▪ - 4.1 μM, Δ - 7.5 μM, and • - 14 μM. The *T_m_* of the ligand-free Hsp90αN component is omitted for clarity. The leftmost data points are controls with no radicicol added. Lines are drawn according to Equation (12) and represent the best possible fit of the data. The solid line is for 4.1 μM, the dashed line for 7.5 μM, and the dotted line for 14 μM protein.

Plots of the melting temperature as a function of added ligand at three protein concentrations are shown in Figure 4. As protein concentration increased, the melting temperature of both the free and bound protein decreased by the same amount. Therefore, the resultant dissociation constant is independent of the protein concentration.

**Figure 5.**
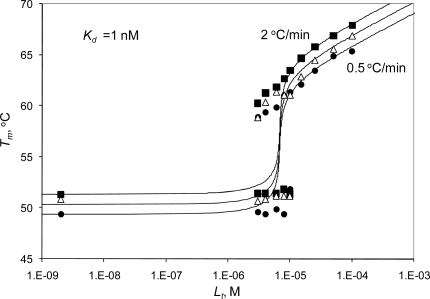
The dependence of Hsp90αN melting temperature on radicicol concentration with various rates of heating. Data points show the melting temperatures of ligand-bound and free components at three heating rates: ▪ - 2 ºC/min, Δ - 1 ºC/min, and • - 0.5 ºC/min. Lines are drawn according to Equation (12). Note that the *T_m_* values obtained at different heating rates yield the same binding constant.

The melting temperatures obtained at various heating rates are shown in Figure 5. Faster heating yielded slightly elevated melting temperatures. The elevation was similar in the absence and in the presence of all added radicicol concentrations. Therefore, the relative increase due to radicicol addition was independent of the rate of heating in the experiment. The radicicol dissociation constant obtained at all three heating rates was 1 nM.

The thermal shift assay helped conclusively determine the Hsp90-radicicol dissociation constant to be about 1 nM, while several ITC approaches yielded the values between 10 and 0.01 nM. Application of both methods is important where greater precision in the determination of the dissociation constant is important.

### 2.4. Discussion

The determination of ligand binding constants ranging above 10^8^ M^−1^ is a difficult task. Isothermal titration calorimetry cannot accurately determine binding constants in the single digit nanomolar range or below by direct titration, and displacement titration of a weakly binding ligand is required [2]. S. Mark Roe *et al*. [31] determined the *K_d_* of radicicol binding to full length and the N-terminal domain of yeast Hsp90 by ITC to be 19 and 2.7 nM, respectively. We believe that these measurements could slightly underestimate the binding constant of radicicol and ethoxzolamide when compared to the thermal shift assay determinations presented here. The *K_d_* of radicicol binding to human Hsp90αN obtained by TSA was equal to 1 nM. Direct comparison to the literature data, however, is not valid since we used recombinantly produced human Hsp90, not the yeast protein.

The binding constant of ethoxzolamide to hCAII determined here by TSA (*K_d_*=2 nM) is greater than the inhibition constant determined by enzyme inhibition methods (*K_i_*=8 nM) [41]. This discrepancy could be due to different experimental conditions, the different nature of the methods, and the experimental error of both methods. The thermal shift assay results depend on the accuracy of the enthalpy of unfolding measurements. The enthalpies of bCAII unfolding were similar when determined by three methods: DSC, TSA, and ITC by acid-denaturation [42]. The enthalpies of Hsp90αN, however, were quite different when measured by TSA and DSC. This indicates that the enthalpies of protein unfolding could be poorly determined by TSA as compared to DSC.

Determination of binding constants by observing thermal denaturation of the protein at subsaturating ligand concentrations was proposed by Brandts and Lin [10]. However, they carried out differential scanning calorimetry experiments rather than observing the fluorescence of ANS as a function of protein denaturation by temperature. The appearance of two transitions in the DSC scans was seen experimentally at very high ribonuclease A concentrations (above 1 mM) in the presence of half the concentration of 2’CMP (above 0.5 mM) [10]. Similar results were obtained with HSA at subsaturating concentrations of aliphatic ligands [20]. Our approach is similar, but experimental data can be obtained more rapidly with fluorescence rather than with DSC. Furthermore, TSA consumes significantly less material than DSC and can be applied in a high-throughput plate format.

Our previous study of carbonic anhydrase using ThermoFluor® (TSA) [17] supported the model described by Equation (12). The denaturation transitions at various protein concentrations with subsaturating ligand concentrations did not show a clear split into two melting transitions. In this study, however, the split was visible from about 10 to 90% saturation with both ethoxzolamide and radicicol. We suppose that for the split to be observable, either the binding constant should be sufficiently tight or both the protein and ligand concentrations should be sufficiently high [10]. In this study, the binding constant was in the order of 10^9^ M^−1^, while in our previous study it was below 10^8^ M^−1^ [17]. Observation and fitting of the split transition provide an opportunity to determine the concentrations of the binding partners, both the protein and the ligand. The binding stoichiometry could be determined with higher precision than that determined in the absence of two transitions.

The entire phenomenon may also depend on the kinetics of binding and denaturation, the rate of heating, and the reversibility of the processes. The process of protein denaturation was dependent on the protein concentration and on the rate of heating, indicating that full equilibrium was not achieved in each experiment. The unfolded protein probably aggregated. However, the demonstration that the binding constant determined did not depend on the protein concentration or the rate of heating confirms the validity of the method in determining tight binding constants.

The application of the TSA method to two unrelated protein-ligand pairs where ligand-free and ligand-bound protein fractions denature separately further extends the applicability of this method for the determination of tightly binding ligand dissociation constants.

## 3. Experimental Section

### 3.1. Materials

Radicicol was purchased from A.G. Scientific, Inc., dissolved in DMSO at 50 mM, and stored at −20 ºC. Radicicol concentration was determined spectrophotometrically using an extinction coefficient of 14,700 M^−1^cm^−1^ in methanol at 265 nm [43]. Partial degradation of radicicol in DMSO solution occurred if stored over a month, especially in the presence of reducing agents. It was important to prepare fresh solutions for all measurements. Ethoxzolamide was purchased from Aldrich (Milwaukee, WI, USA).

### 3.2. Hsp90 constructs

The gene encoding full-length human Hsp90α protein was purchased from RZPD, Deutsches Ressourcenzentrum für Genomforschung GmbH (Germany). For protein expression, the *N*-terminal fragment of the Hsp90α gene, corresponding to amino acids 3–241, was inserted into the pET-15b vector (Novagen, Madison, WI, USA) using XhoI and BamHI restriction sites, fusing a His_6_ tag to the *N*-terminus of the protein.

### 3.3. Protein expression and purification

His_6_-tagged Hsp90αN protein was expressed in the *Escherichia coli* strain BL21 (DE3). Bacterial cultures were grown until an A_550_ of 0.5 – 0.8 was reached and the expression was induced by the addition of 1 mM isopropyl-1-thio-β-d-galactopyranoside (final concentration). Protein was purified according to [44]. Cells were lysed by sonication. Soluble protein was purified using a Ni-IDA affinity column, followed by an anion exchange chromatography column (Amersham Biosciences). Eluted protein was dialyzed into a storage buffer containing 20 mM Tris (pH 7.5), 50 mM Na_2_SO_4_, and 1 mM DTT. The purity of the Hsp90αN preparations was analyzed by SDS-PAGE and determined to be higher than 98%. Protein concentrations were determined by UV-VIS spectrophotometry and confirmed by standard Bradford methods. Protein stock solution was stored at −70 ºC.

### 3.4. Production of recombinant human carbonic anhydrase II

Recombinant human carbonic anhydrase was expressed in *E. coli* and purified as previously described [7].

### 3.5. Mass spectrometry experiments

The recombinantly produced protein molecular weight (MW) was determined by a Q-TOF Ultima Global mass spectrometer (“Micromass,” Manchester, UK), electrospray ionization, positive (ESI+), capillary OD=90 μm, ID=20 μm, injection speed=0.5 μL/min. Protein solution was diluted with the same volume of acetonitrile solution containing 1% formic acid. MS spectra were processed with MassLynx 4.0 software. Solutions of Hsp90αN with radicicol were incubated under the same conditions as the solutions used for protein denaturation experiments. Hsp90αN solutions were then analyzed by MS to test for covalent modifications of Hsp90αN by radicicol, since it was reported that in the presence of high concentration of reductants such as dithiothreitol (DTT), radicicol was unstable and covalently modified [38].

### 3.6. Isothermal titration calorimetry

The protein solution (2–5 μM) was loaded into the VP-ITC isothermal titration calorimeter (Microcal, Inc.) cell (about 2 mL, cell volume about 1.4 mL). The titration syringe (250 μL volume) was filled with 20 to 50 (usually 40) μM ligand solution. Titrations were carried out using 25 injections of 10 μL each, injected at 3 to 4 minute intervals. Stirring speed was 400 rpm. Titrations were carried out at constant temperature in the 13 – 37 ºC temperature range. Ligand solution was prepared in the same buffer as protein solution containing the same concentration of DMSO (usually 1%).

### 3.7. Protein denaturation experiments

The thermal shift assay was performed using the iCycler iQ Real Time Detection System (Bio-Rad, Hercules, CA), originally designed for PCR, and the ISS PC1 spectrofluorimeter with a temperature-controlled water bath. The temperature was measured inside the cuvette using a TFN520 thermometer (Ebro Electronic GmbH & Co KG, Germany) with an error of ±0.2 ºC. Protein concentrations were measured spectrophotometrically (ɛ_280_ (Hsp90αN)=15,930 M^−1^cm^−1^, ɛ_280_ (hCAII)=50,420 M^−1^cm^−1^). Protein and ligand concentrations and buffers are shown in the figure legends. DMSO was added to make up 1% (v/v) of the solution in each measurement. Protein unfolding was monitored by measuring the fluorescence of the solvatochromic fluorescent dye Dapoxyl™ sulfonic acid sodium salt (iCycler) or ANS (ISS PC1), both at 50 μM. The total volume of the reaction was 0.01 mL (iCycler) or 3 mL (ISS PC1) using a covered cuvette to prevent evaporation. Samples in the iCycler were overlayed with 2.5 μL of silicone oil DC 200. The assay was performed in 96-well iCycler iQ PCR plates. Samples in ISS PC1 were excited with UV light of 380±5 nm, and the ANS fluorescence emission monitored at 510±5 nm. Heating of the samples was carried out at a speed of 0.5, 1, or 2 ºC/min.

### 3.8. Differential Scanning Calorimetry (DSC) experiments

DSC experiments were carried out using an MC-2 Scanning Calorimeter (MicroCal, Inc. North Hampton, MA). Hsp90 samples contained 120 μM Hsp90αN, 0 to 360 μM radicicol, 50 mM Hepes buffer, pH 7.5, and 100 mM NaCl. Carbonic anhydrase samples contained 100 μM hCAII, 0 to 1 mM ethoxzolamide, 50 mM sodium phosphate (pH 7.0), 50 mM NaCl, and 2% DMSO (cuvette volume 1.2 mL). A scan rate of 1 ºC /min was applied.

## 4. Conclusions

The thermal shift assay is a good way to measure tight ligand-protein binding constants. The assay can be performed in high-throughput fashion using a plate format even at subsaturating ligand concentrations. It is important to check and confirm ITC results of tight binding reactions by an alternative method such as TSA.

## Figures and Tables

**Table 1.** Thermodynamic parameters of free and ligand-bound unfolding of Hsp90αN (14 μM) and hCAII obtained by fitting to the data in Figure 2.

[Ligand], μM	*T_free_*, ºC	*T_bound_*, ºC	Δ*_u_H_free_*, kJ/mol	Δ*_u_H_bound_*, kJ/mol	*n*
Uncertainties-	±1.2	±1.2	±140(TSA) ±30 (DSC)	±140 (TSA) ±30 (DSC)	±0.15
Hsp90αN (14 μM) with radicicol (TSA)					
0	51.6	–	670	–	0
2	52.7	61.1	650	610	0.15
3	52.5	61.0	610	610	0.22
6	53.0	61.8	600	600	0.44
10	51.4	63.6	610	610	0.74
20	–	64.9	–	870	1
50	–	66.9	–	900	1
Hsp90αN (120 μM) with radicicol (DSC)					
0	48.2	–	290	–	0
60	48.6	63.4	302	283	0.5
360	–	66.9	–	477	1
hCAII (5 μM) with ethoxzolamide (TSA)					
0	57.8	–	690	–	0
1.5	58.1	63.9	720	750	0.3
2	58.8	64.6	660	720	0.4
3	59.8	64.6	610	740	0.6
50	–	68.2	–	780	1
hCAII (100 μM) with ethoxzolamide (DSC)					
0	56.5	–	570	–	0
22	56.5	66.5	490	890	0.2
50	56.4	66.9	420	860	0.5
1000	–	69.4	–	790	1

Parameters used to fit Equation (10): Δ*_u_C_p_*=15 kJ×mol^−1^K^−1^ (Hsp90αN) and 17 kJ×mol^−1^K^−1^ (hCAII), *a_F_*=4300–6500 (Hsp90αN) and 2100–4500 (hCAII) arbitrary fluorescence units, *b_F_*=−73 (Hsp90αN) and −50 (hCAII), *c_F_*=0.7 (Hsp90αN) and 0.3 (hCAII), *a_U_*=24300–24600 (Hsp90αN) and 19000–27000 (hCAII) arbitrary fluorescence units, *b_U_*=−479 (Hsp90αN) and −300 (hCAII), *c_U_*=2.7 (Hsp90αN) and 2.0 (hCAII).

## Abbreviations:

ANS: 1,8-anilinonaphthalene sulfonate;
DMSO: dimethyl sulfoxide;
DSC: differential scanning calorimetry;
DTT: 1,4-dithiothreitol;
bCAII: bovine carbonic anhydrase II;
hCAII: recombinant human carbonic anhydrase II;
Hsp90αN: *N*-terminal domain of recombinant human alpha Hsp90;
Hsp90: heat shock protein 90;
ITC: isothermal titration calorimetry;
Rdc: radicicol;
Tris: tris(hydroxymethyl)-aminomethane;
TSA: thermal shift assay
